# A Case of Gorlin-Goltz Syndrome Presented With Multiple Odontogenic Keratocysts in the Jaw Without Skin Manifestation

**DOI:** 10.7759/cureus.24666

**Published:** 2022-05-02

**Authors:** A Rupesh Rao, Amar Taksande

**Affiliations:** 1 Pediatrics, Jawaharlal Nehru Medical College, Wardha, IND

**Keywords:** autosomal dominant, keratocysts, basal epitheliomas, ptch, gorlin-goltz syndrome

## Abstract

Gorlin-Goltz syndrome is a hereditary autosomal dominant condition with high penetrance and varied phenotypic expressiveness that can appear spontaneously. It is estimated that between 30% and 50% of people with this disease do not know if any of their family members have had it. Patched (PTCH), a tumor suppressor gene found on the 9q22.3 chromosome, has been identified as the cause of Gorlin-Goltz syndrome. This case emphasizes the necessity of awareness of this uncommon illness in young people who do not have any skin blemishes. Due to the severity of clinical manifestations, early identification of the illness and a long follow-up time are critical.

Furthermore, a multidisciplinary team consisting of a dentist, dermatologist, geneticist, and neurologist, is necessary to improve overall survival rates. Gorlin-Goltz syndrome is inherited as an autosomal dominant disease. In almost 50% of cases, many people do not know whether they have a positive family history. It is not always present with basal cell epitheliomas or skin manifestation.

## Introduction

Gorlin-Goltz syndrome is a rare multisystemic illness inherited as autosomal dominant, with a high level of penetrance and variable expression [1]. This illness has been given several names throughout the years, including "basal cell nevus syndrome" [2], "nevoid basal cell carcinomas syndrome", "multiple basal epitheliomas, jaw cysts, and bifid rib syndrome" [3]. Jarisch and White gave the primary description of this syndrome in 1894. They focused on the presence of multiple basocellular carcinomas. Then, Straith, in the year 1939, also reported a case where he diagnosed multiple basocellular carcinomas along with the cyst. Gross, 1953, reported a similar case with superadded findings like synostosis of the first rib and the bilateral bifurcation of the sixth rib. Around the same period, Ward and Bettley also associated the presence of plantar and palmar pits with this condition. However, until 1960, Gorlin and Goltz formed a triad that would help and characterize the diagnosis of this condition, namely, kerotocysts in the jaw, bifid ribs, and the multiple basocellular epitheliomas. This triad was later modified by Rayner et al.. They established that for the diagnosis, at least cysts had to appear in combination with calcification of the falx cerebri or palmar and plantar pits [1]. The incidence of the Gorlin-Goltz syndrome is estimated at 1 in 50,000 to 150,000 in the general population. Farndon et al. reported a minimum prevalence of 1 in 57,000 people [4]. Shanley et al. in Australia and Lo Muzio et al. in Italy estimated the prevalence as 1 per 64,000 and 256,000, respectively [5-6]. Evans et al. reported that the prevalence rate in the United Kingdom was 1 per 560,000 [7].

## Case presentation

**Figure 1:**
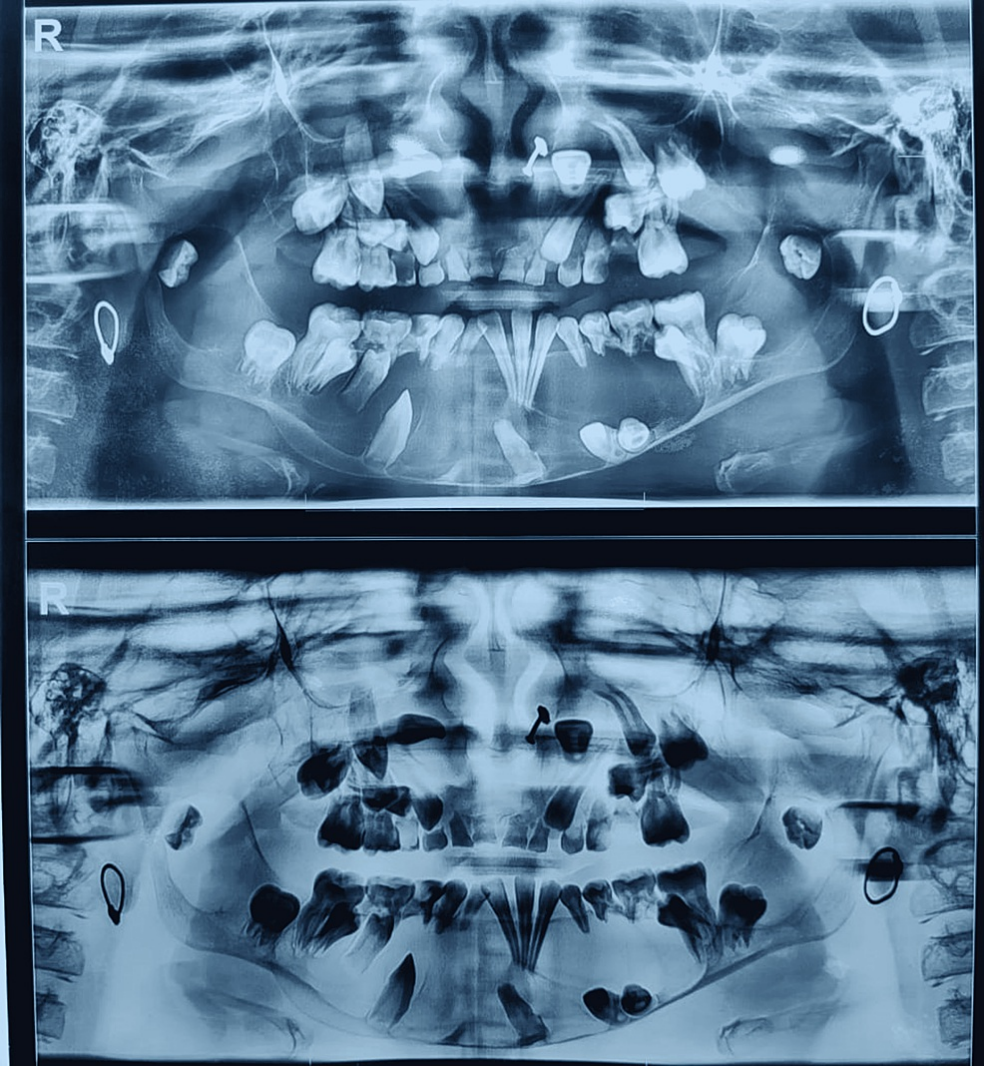
Orthopantomogram shows lytic lesion and malocclusion.

A 13-year-old female child came with chief complaints of swelling of the bilateral lower jaw on the backside for two months which was initially small in size and gradually progressed to the present size, which was 4 X 3cm. The child also had difficulty eating because she could not open her mouth completely. With these complaints, the patient came to our hospital for further management. In our hospital first Orthopantomograph was done, which show showing multiple jaw cysts with impacted and unerupted teeth (figure 1).

**Figure 2:**
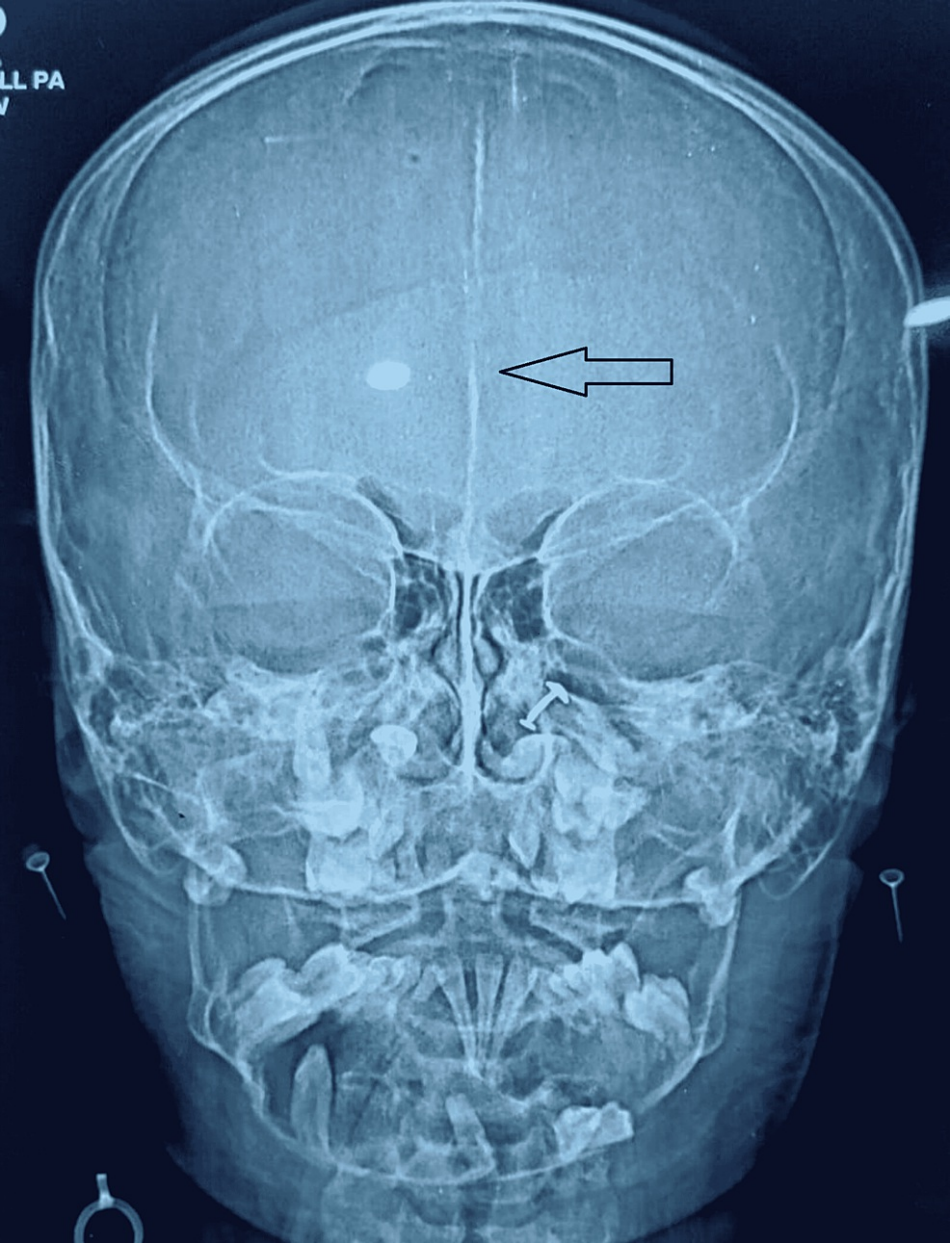
X-ray showing Falx cerebrum calcification.

**Figure 3:**
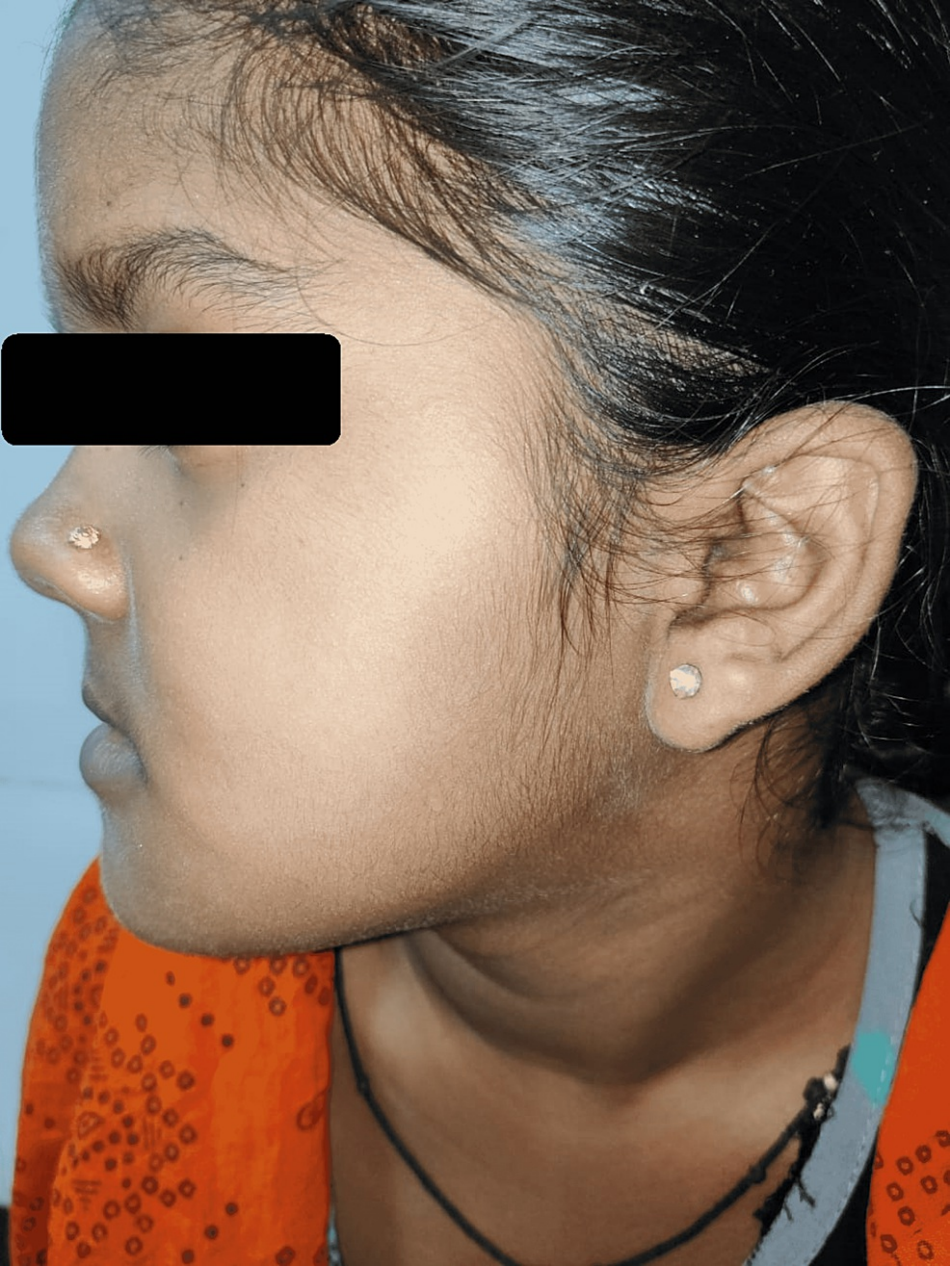
Dysmorphic face

**Figure 4:**
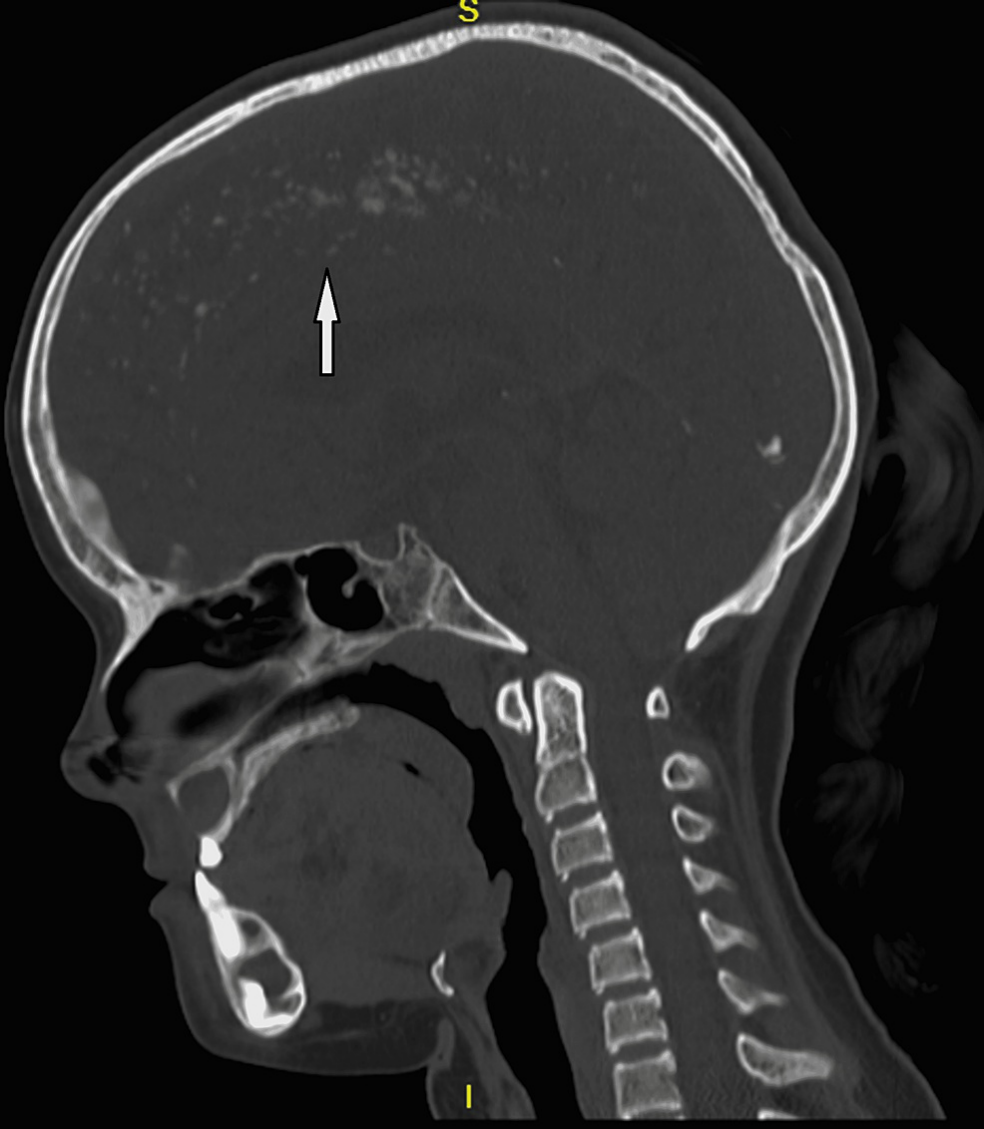
CT shows calcification in falx cerebri and tentorium cerebellum

**Figure 5:**
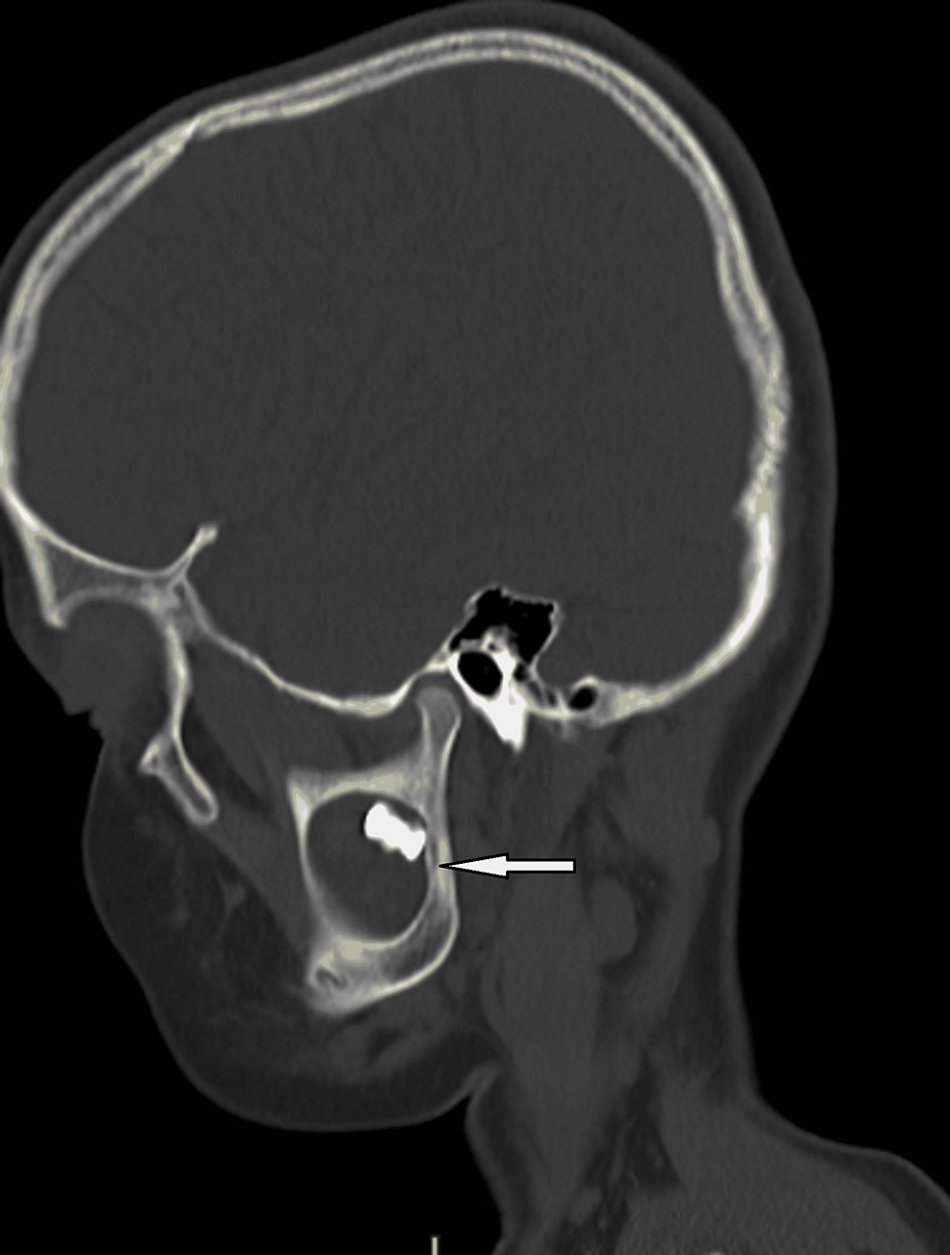
CT scan shows a lytic lesion with a tooth.

For further evaluation, a CBCT scan was done, which showed a lytic lesion in the mandible, and which biopsy was done, which showed histological features suggesting odontogenic keratocyst. With suspicion of Gorlin-Goltz syndrome in mind, we look for other associated findings. On x-ray skull, we found that child have falx cerebri calcification (figure 2). On general examination, the facial deformity is present (figure 3). On a CT scan of the head, we found that child has calcification in falx cerebri and tentorium cerebelli (figure 4). It also shows an expansile cystic lesion in the mandible with a tooth inside it (figure 5). There is no positive family history of similar complaints in the family. Later child was shifted to the operation theatre for enucleation, and curettage was done.

## Discussion

Clinically this condition is characterized by different signs and symptoms. Diagnosis is based on the most frequent and specific features of the syndrome, as given by Evans et al. in 1993 [7]. Gorlin-Goltz syndrome might be diagnosed when two major or one significant and two minor criteria are present.

The major criteria are multiple basal cell carcinoma or one occurring under the age of 20 years, Histologically proven Odontogenic keratocyst (OKC) of the jaws, Palmar or plantar pits (three or more), Bilamellar calcification of the falx cerebri, bifid, fused, or markedly splayed ribs, First-degree relative with Nevoid Basal Cell Carcinoma syndrome.

The minor criteria are macrocephaly (adjusted for height), Congenital malformation: cleft lip or palate, frontal bossing, coarse face, moderate or severe hypertelorism, Other skeletal abnormalities: Sprengel deformity, marked pectus deformity, marked syndactyly of the digits, Radiological abnormalities: bridging of the sella turcica, vertebral anomalies such as hemivertebrae, fusion or elongation of the vertebral bodies, modeling defects of the hands and feet, or flame-shaped hands or feet, Ovarian fibroma, Medulloblastoma.

In our patient, the diagnosis of the Gorlin-Goltz syndrome was established by the presence of 4 primary criteria (multiple odontogenic keratocysts, bifid rib, palmar pits, and calcified falx cerebri) and one minor criterion (frontal bossing). Other symptoms of this condition are seen in less than ten percent of individuals with multiple OKCs. It has therefore been suggested that multiple OKCs alone may be confirmatory of the syndrome [8]. Multiple cystic lesions affecting the mandible were also seen in our case, which was histopathologically classified as an odontogenic keratocyst.

The OKC is now termed a 'keratocystic odontogenic tumor' (KCOT) [9]. In the form of numerous cystic lesions, it may be linked to the Gorlin-Goltz syndrome. Despite its bland histological appearance, the KCOT is locally damaging. Enucleation may be regarded appropriate treatment for OKCs if all teeth involved in or in contact with the disease are removed. Depending on the extent of the lesion and the patient's age, this therapy can be supplemented with fenestration or open packing. It would be challenging to choose invasive surgery if the patient is in their first or second decade of life with previously unerupted permanent teeth involving OKCs. The guidelines for follow-up of NBCCS as given by de Amezaga et al. [1] in table 1.

**Table 1:** The guidelines for follow-up of NBCCS. Reference: Amezaga et al. [1]

Neurological examination - twice yearly
Cerebral MRI - once a year for 1 to 7 years of age
Skin examination - yearly
Cardiologic examination - according to signs and symptoms
Genetic counseling of families as it is an autosomal dominant disorder.

## Conclusions

This case highlights the necessity for young individuals without skin blemishes being aware of this rare syndrome. Due to the severity of clinical manifestations, early identification of the illness and a long follow-up time are critical. Furthermore, a multidisciplinary team consisting of a geneticist, dentist, dermatologist, and neurologist, is necessary to improve overall survival rates. So, one of Gorlin-Goltz syndrome's major abnormalities, OKCs, was recognized and treated, whereas the other defects did not require active care.
